# Unique composition of ocular surface microbiome in the old patients with dry eye and diabetes mellitus in a community from Shanghai, China

**DOI:** 10.1186/s12866-023-03176-2

**Published:** 2024-01-10

**Authors:** Zhangling Chen, Senlin Lin, Yi Xu, Lina Lu, Haidong Zou

**Affiliations:** 1 Department of Ophthalmology, Shanghai General Hospital, Nanjing Medical University, No. 100, Haining Road, Hongkou District, Shanghai, 200080 China; 2 Department of Ophthalmology, Songjiang Hospital Affiliated to Shanghai Jiao Tong University School of Medicine, Shanghai, China; 3 Shanghai Eye Diseases Prevention and Treatment Center/Shanghai, Eye Hospital, Shanghai, China; 4 Department of Ophthalmology, Shanghai General Hospital, School of Medicine, Shanghai Jiao Tong University, Shanghai, China; 5 Shanghai Key Laboratory of Fundus Diseases, Shanghai, China; 6 National Clinical Research Center for Eye Diseases, Shanghai, China; 7 Shanghai Engineering Center for Precise Diagnosis and Treatment of Eye Diseases, Shanghai, China

**Keywords:** Diabetes, Dry eye, Ocular surface, Microbiome, 16S rRNA

## Abstract

**Background:**

This study investigates the variations in microbiome abundance and diversity on the ocular surfaces of diabetic patients suffering from dry eye within a community setting. The goal is to offer theoretical insights for the community-level prevention and treatment of dry eye in diabetic cohorts.

**Methods:**

Dry eye screening was performed in the Shanghai Cohort Study of Diabetic Eye Disease (SCODE) from July 15, 2021, to August 15, 2021, in the Xingjing community; this study included both a population with diabetes and a normal population. The population with diabetes included a dry eye group (DM-DE, *n* = 40) and a non-dry eye group (DM-NoDE, *n* = 39). The normal population included a dry eye group (NoDM-DE, *n* = 40) and a control group (control, *n* = 39). High-throughput sequencing of the 16 S rRNA V3-V4 region was performed on conjunctival swab from both eyes of each subject, and the composition of microbiome on the ocular surface of each group was analyzed.

**Results:**

Significant statistical differences were observed in both α and β diversity of the ocular surface microbiome among the diabetic dry eye, diabetic non-dry eye, non-diabetic dry eye, and normal control groups (*P* < 0.05).

**Conclusions:**

The study revealed distinct microecological compositions on the ocular surfaces between the diabetic dry eye group and other studied groups. *Firmicutes* and *Anoxybacillus* were unique bacterial phyla and genera in the dry eye with DM group, while *Actinobacteria* and *Corynebacterium* were unique bacterial phyla and genera in the normal control group.

**Supplementary Information:**

The online version contains supplementary material available at 10.1186/s12866-023-03176-2.

## Introduction

The evolving lifestyles and environmental changes in contemporary society have led to a rising prevalence of dry eye syndrome (DES), now recognized as a significant public health concern impacting ocular well-being. Dry eye (DE) can cause many discomforting symptoms in the eyes, including eye dryness, increased blinking, foreign body sensation, pain, photophobia, tearing and visual disorders, which may interfere with people’s daily life [1]. The improvement of living standards had led to an increase in the prevalence of diabetes year by year, and with the progression of the disease, the risk of chronic eye diseases increases [2, 3]. Studies have shown that the prevalence of dry eye with diabetes mellitus(DM)in adults is significantly higher than that among healthy people [4–6]. Although the detailed pathogenesis of DES is not completely clear, it is usually accompanied by changes in the quality and quantity of tears and inflammatory reactions on the ocular surface [7]. Increasing research evidence has shown that there is a relationship between DM and DES [8, 9].

In 2008, the National Institutes of Health of the United States initiated the Human Microbiome Project (HMP), which uncovered the presence of highly abundant and diverse microbiome inhabiting the human body [10]. In recent times, there has been a progressive focus on studying the attributes of microbiome residing on the ocular surface, and an emerging research field is focusing on the microbiome of the ocular surface [11, 12]. An increasing number of studies [13–19] have shown that the microbiome significantly influences the well-being and pathogenesis of ocular conditions, thereby holding substantial significance in the realm of eye health. Simultaneously, research has indicated a strong correlation between the microbiome of the eye and DES, with *Staphylococcus aureus*, *coagulase-negative Staphylococcus*, and *Corynebacterium* being associated with the prevalence of DES [20, 21]. In addition, the positive rate of microbial culture was higher in DES-affected eyes, indicating that some microbes were involved in the incidence of DES. Previous study has shown that *Lactobacillus* and *unclassified Clostridium* may be involved in the pathogenesis of DE in hospital patients with DM by 16 S amplicon-sequencing [22]. To understand the ocular surface microbiome association between DM and DES, more evidence is needed.

In contrast to conventional microbial culture methods, molecular biology techniques such as 16 S rRNA sequencing offer a more comprehensive and precise means of identifying the species composition of ocular surface microbiome. While existing research has predominantly concentrated on hospital-based populations with DM-related DE, there remains a notable gap in understanding these conditions within community settings. This study aims to bridge this gap by employing modern genomics detection technology to analyze the ocular surface microbiome in both diabetic and non-diabetic community populations, thereby enriching our understanding of DE’s etiology.

## Materials and methods

During the period from July 15 to August 15, 2021, individuals aged over 60 years in the Xinjing community were systematically screened for diabetic eye diseases. A total of 158 elderly participants, comprising 79 diabetics and 79 non-diabetics, were enrolled and further divided into four distinct groups: DE with DM group (DM-DE, *n* = 40), non-DE with DM group (DM-NoDE, *n* = 39), DE without DM group (NoDM-DE, *n* = 40) and normal control group (Control, *n* = 39). The study subjects were selected based on specific inclusion and exclusion criteria [23]. The inclusion criteria were as follows: (1) subjects who provided informed consent and understood the study’s details; (2) subjects aged over 60 years; (3) subjects capable of cooperating during eye examinations and specimen collection; and (4) subjects diagnosed with diabetes according to the diagnostic criteria set by the World Health Organization (WHO) [24]. According to the International Dry Eye Workshop II (DEWS II) criteria for DE examination and diagnosis [25]. On the other hand, the exclusion criteria encompassed various conditions such as (1) eyelid disorders such as impingement, entropion, or incomplete closure; (2) conjunctival diseases like infectious conjunctivitis, allergic conjunctivitis, pterygium, or conjunctival scarring; (3) history of severe chemical damage or trauma to the eye; (4) recent eye surgery or corneal contact lens wear within the past three months; (5) ongoing treatment with eye drops; and (6) systemic diseases including systemic lupus erythematosus, Sjogren’s syndrome, Grave’s eye disease, among others.

All participants included in the screening were directed to an examination room that provided appropriate lighting, temperature, and humidity for the collection of conjunctival swabs from both eyes. The sampling procedures consisted of the following steps [23]: the patient’s eyelid skin was gently wiped twice using a saline-soaked cotton swab, followed by the utilization of a conjunctival swab. Different sides of the swab were used to wipe the upper and lower conjunctival sacs of both eyes, and the swab head was retained as the specimen (Supplementary Material 1). No eye drops, including anesthetics, were applied to the eyes before the conjunctival swab was taken. The entire process was conducted delicately to ensure minimal discomfort and avoid triggering tear secretion. Subsequently, the specimens were promptly placed in tubes containing a DNA protective solution and stored in a freezer at -20 °C. DNA extraction was carried out immediately after the collection of all specimens.

All participants underwent eye examinations and specimen collection at Xinjing Community Health Center according to a prearranged schedule. The collection of ocular specimens was performed by a trained ophthalmologist, Z.C., to ensure consistent and standardized results.

For the assessment of sample size, micropower [26] was utilized, a power and paired sample size estimator based on the permutational multivariate analysis of variance (PERMANOVA) application. Following a similar approach to a previous study on ocular microbiome analysis using low abundance 16 S rRNA datasets [27], a minimum sample size of 30 was determined to achieve a discriminant power of 0.8 at a significance level of 0.05. Thus, we aimed to enroll a minimum of 35 subjects per study group, with 40 subjects in the DM-DE group, 39 subjects in the DM-NoDE group, 40 subjects in the NoDM-DE group, and 39 subjects in the Control group, meeting the required sample size for the study. Statistical analysis was conducted using SPSS 22.0 software. The ages of the four groups of adults were compared using one-way analysis of variance, while sex comparisons were performed using the chi-square test. Statistical significance was considered for P-values less than 0.05.

## Microbiological testing and data analysis

The conjunctival swab specimens collected were subjected to DNA extraction following the instructions provided by the kit manufacturer. Total microbial genomic DNA was extracted from all specimens using the OMEGA Soil DNA Kit (M5635-02) from Omega Bio-Tek (Norcross, GA, USA) [23, 28]. Subsequently, the extracted genomic DNA was stored in a freezer at -20 °C for subsequent analysis. The quality and quantity of DNA were assessed through agarose gel electrophoresis and a NanoDrop NC-2000 spectrophotometer, respectively. For amplification of the V3-V4 region of the bacterial 16 S rRNA gene, PCR was performed using the forward primer 338 F (5’-ACTCCTACGGGAGGCAGCA-3’) and the reverse primer 806R (5’-GGACTACHVGGGTWTCTAAT-3’). Following amplification, equal amounts of the resulting PCR products were pooled together, and double-end 2 × 250 bp sequencing was conducted on the Illumina MiSeq platform using the MiSeq Reagent Kit v3 program at Personal Biotechnology Co., Ltd (Shanghai,China).

Sequence data analysis was conducted using QIIME2 2019.4 and the R package (v3.2.0). The Greengenes database served as a reference for comparing characteristic amplicon sequence variant (ASV) sequences, enabling the retrieval of taxonomic information associated with each ASV. ASVs with abundance values below 0.001% of the total sequencing across all samples were excluded. The ASV abundance matrix was randomly subsampled at various depths, and a sparse curve was generated to depict the number of sequences sampled at each depth alongside their corresponding ASVs. To address sequencing depth-induced diversity variations among samples, the ASV abundance matrix was rarified to the lowest 95% of the sequences present in all samples. Alpha (α) diversity and beta (β) diversity were analyzed. α diversity and β diversity represent the diversity of a species within its habitat and between habitats respectively, to comprehensively evaluate its overall diversity. Box line plots were employed to compare the richness and evenness of ASVs across different sample groups. β diversity analysis utilizing the UniFrac distance metric was performed to explore alterations in microbial community structure between samples. Principal coordinate analysis (PCoA) and nonmetric multidimensional scaling analysis (NMDS) were utilized to visualize species composition profiles at the genus level. PERMANOVA was employed to assess the significance of differences in microbial community structure between groups. Furthermore, the linear discriminant analysis effect size (LEfSe) method, known as LDA effect size, was applied to identify taxonomic units that exhibited significant differences between groups.

## Results

### Basic information

The basic information of the 158 subjects is shown in Table 1, and there was no statistically significant difference in sex or age (*P* > 0.05).

**Table 1:** Basic information of 158 subjects

	Group				x^2^ or F	P
	DM-DE	DM-NoDE	NoDM-DE	Control		
Gender (male/female)	18/22	21/18	18/22	16/23	*x*^*2*^ = 1.484	*P* = 0.709
Age (year)	67.90 ± 6.52	67.03 ± 6.04	67.70 ± 4.08	68.05 ± 4.37	*F* = 0.279	*P* = 0.841

### High-quality sequences and ASVs/OTUs

**Fig. 1:**
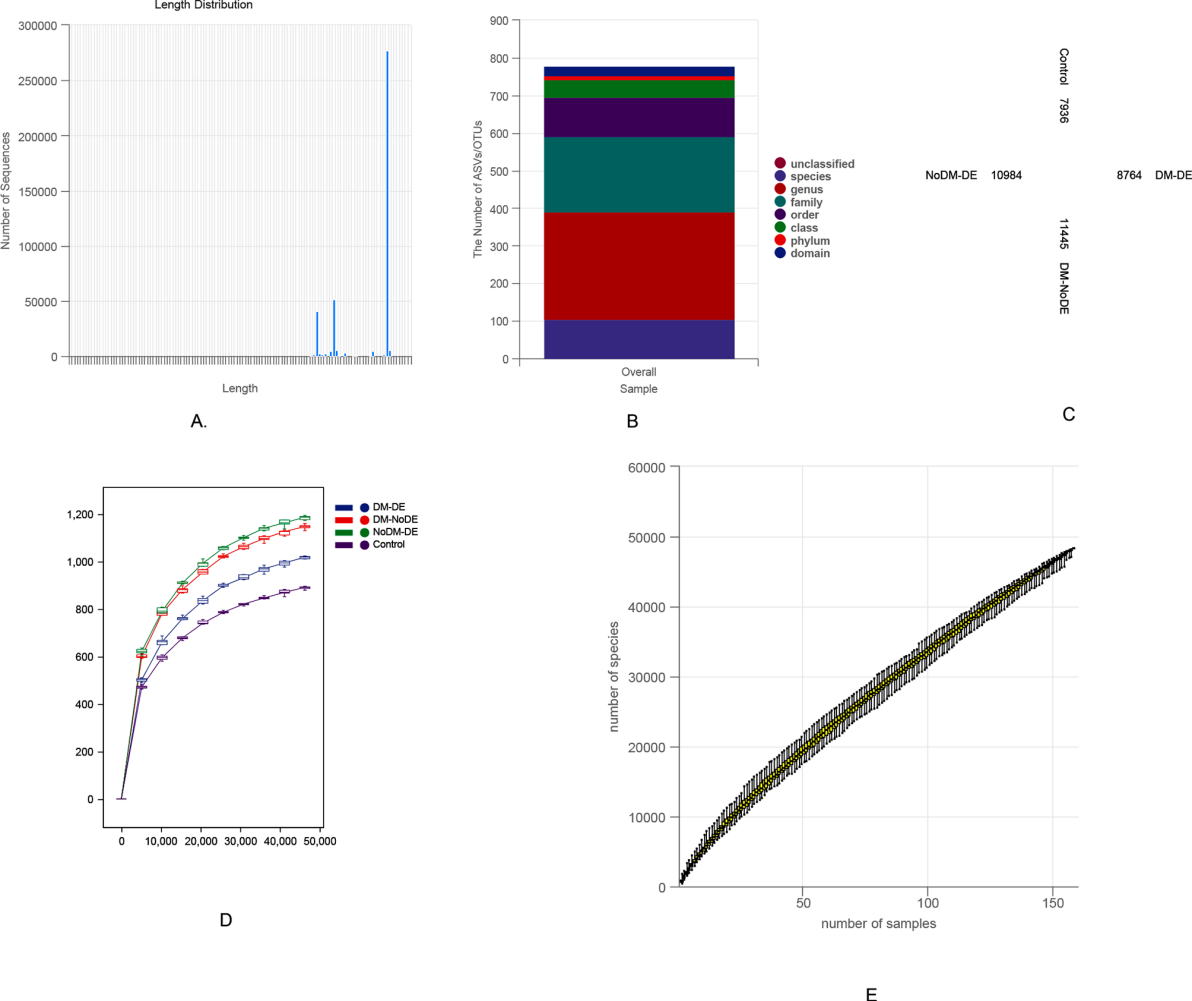
(**A**) Sequence length distribution; (B) the number of ASVs/OTUs; (**C**) ASV/OUT Venn diagram; (**D**) Rarefaction curve; (**E**) Species accumulation curves

A total of 16,022,648 (reads) high-quality sequences were obtained by using the DADA2 method to remove primers, quality filtering, and removal of chimeras, with an average of 101,409 (reads) high-quality sequences per specimen. Among all the specimens, the maximum number of sequences was 144,325 (reads), and the minimum number of sequences was 48,760 (reads). The average length of each sequence was 424 bp (base pair, bp), and 99.9% of the high-quality sequences ranged from 400 to 431 bp. The high-quality sequences were aggregated into 780 operational taxonomic units (OTUs) representing 387 individual species belonging to 127 genera, 92 families, 58 orders, 39 classes, and 19 phyla. A total of 1,986 OTUs were recorded in all groups, indicating the presence of the core microbiome of the ocular surface (Fig. 1–A, B, C).

### Evaluation of sequencing depth and sample size

The shape of the sparsity curve provided insights into the impact of sequencing depth on microbial community diversity. In our study, the sparsity curve demonstrated a gradual plateau as the amount of sequencing data increased, suggesting that the current sequencing depth adequately captured the richness and evenness of microbiome within the sample. The species accumulation curve indicated that the sample size was appropriate for the study (Fig. 1D and E).

### α diversity analysis and β diversity analysis

**Fig. 2:**
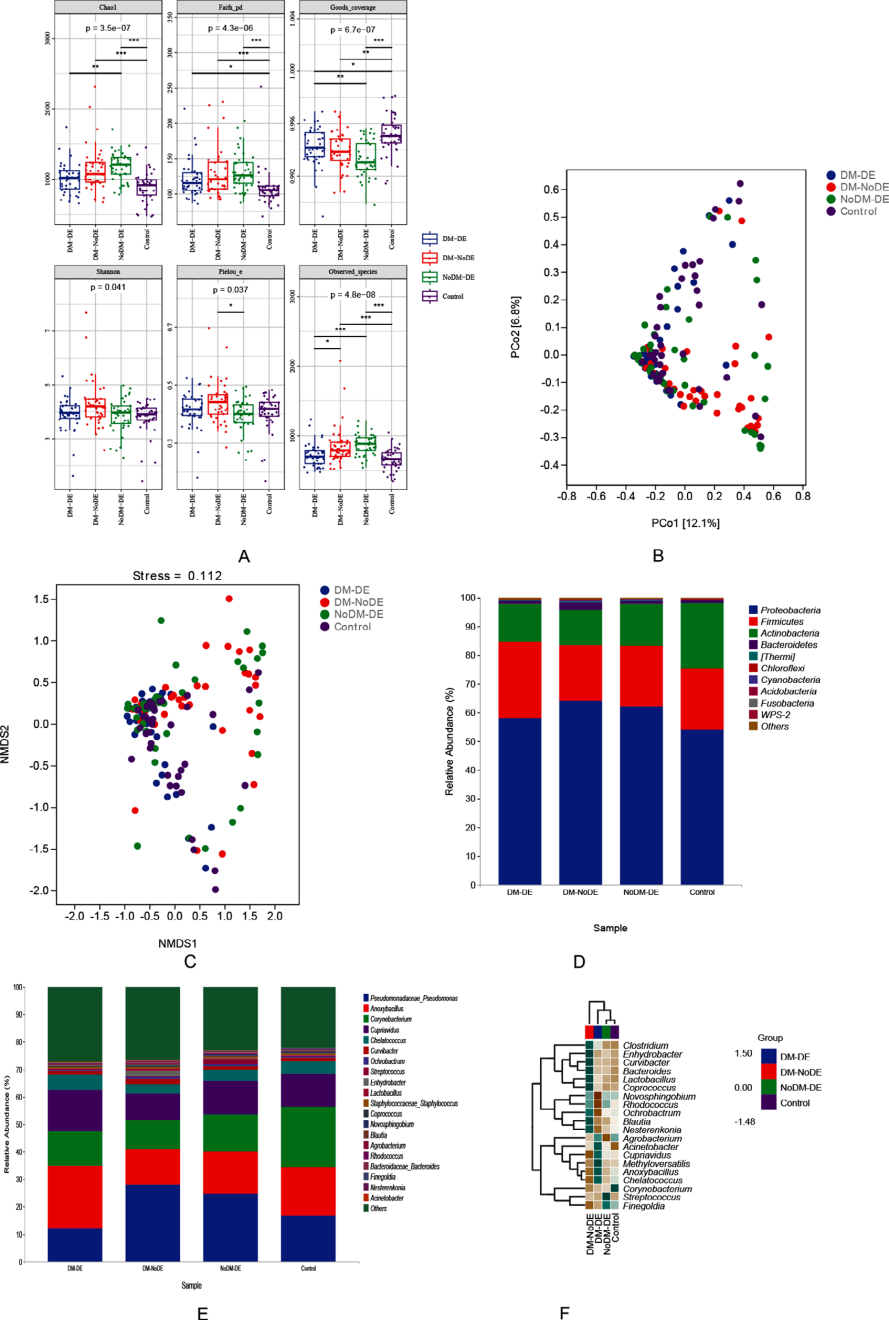
(**A**) α-Diversity index analysis of the four groups; (**B**) PCoA analysis; (**C**) NMDS analysis; (**D**) the relative abundance of four groups at phyla level (top 10); (**E**) the relative abundance of four groups at genus level (Top 20); F Species composition heatmap at the genus level (Top 20)

Richness was represented by Chao1 and the observed species index, diversity was represented by the Shannon index, evolution diversity was represented by Faith’s PD index, evenness was represented by Pielou’s evenness index, and the coverage diversity was represented by Good’s coverage. The results of these α diversity indices showed that the ocular surface microbiome in the NoDM-DE group had higher richness, and the ocular surface microbiome in the diabetes group had more diversity and uniformity. The t-test showed significant differences among all groups (Fig. 2A).

A partial least squares discriminant analysis (PLS-DA) model was established using the relative abundance data at the species level. The Bray-Curtis distance algorithm was employed, with a two-dimensional NMDS representation and a 0.95 elliptic confidence. The results of the principal coordinate analysis (PCoA) and NMDS analysis are shown in Fig. 2B and C. The species composition varied significantly between the groups, indicating distinct differences.

### Microbiological taxonomic analysis

The bacterial composition of the ocular surface in the four groups was compared, and the 16 S rRNA sequences of individual bacteria were classified at the phylum and genus levels. At the phylum level (Fig. 2D), the 16 S rRNA gene sequencing of ocular surface bacteria in the four groups revealed the presence of four significant phyla: *Proteobacteria*, *Firmicutes*, *Actinobacteria*, and *Bacteroidetes*. The abundance of *Proteobacteria* in the DM-DE group (58.00%) was higher compared to the control group (53.98%). In the DM-DE group, the abundance of *Firmicutes* (26.66%) was significantly higher than that in the other groups (DM-NoDE group: 19.40%, NoDM-DE group: 21.16%, control group: 21.44%). The control group exhibited the highest abundance of *Actinobacteria* (22.78%) compared to the DM-DE group (13.30%), DM-NoDE group (12.10%), and NoDM-DE group (14.49%). *Bacteroidetes* exhibited the highest abundance in the DM-NoDE group (2.58%) compared to the DM-DE group (1.03%), NoDM-DE group (1.06%), and control group (0.95%).

At the genus level, the majority of the 16 S rRNA gene sequencing results from the four groups of ocular surface bacteria were assigned to 20 genera (Fig. 2E). These genera included *Pseudomonas, Anoxybacillus, Corynebacterium, Cupriavidus, Chelatococcus, Curvibacter, Ochrobactrum, Streptococcus, Enhydrobacter, Lactobacillus, Staphylococcus, Coprococcus, Novosphingobium, Blautia, Agrobacterium, Rhodococcus, Bacteroides, Finegoldia, Nesterenkonia*, and *Acinetobacter.* In the DM-DE group, *Anoxybacillus, Cupriavidus*, and *Chelatococcus* were significantly more abundant compared to the other groups, while *Pseudomonas* exhibited significantly lower abundance than in the other groups. The control group had the highest abundance of *Corynebacterium* (Fig. 2F).

In all samples, the microbiome with abundances greater than 1% were clustered at the phylum and genus levels, among which, *Proteobacteria*, *Firmicutes*, *Actinobacteria* and *Bacteroidetes* were the dominant bacterial phyla, and *Pseudomonas, Anoxybacillus, Corynebacterium, Cupriavidus, Chelatococcus*, and *Curvibacter* were the dominant bacterial genera.

### Analysis of differences between groups

**Fig. 3:**
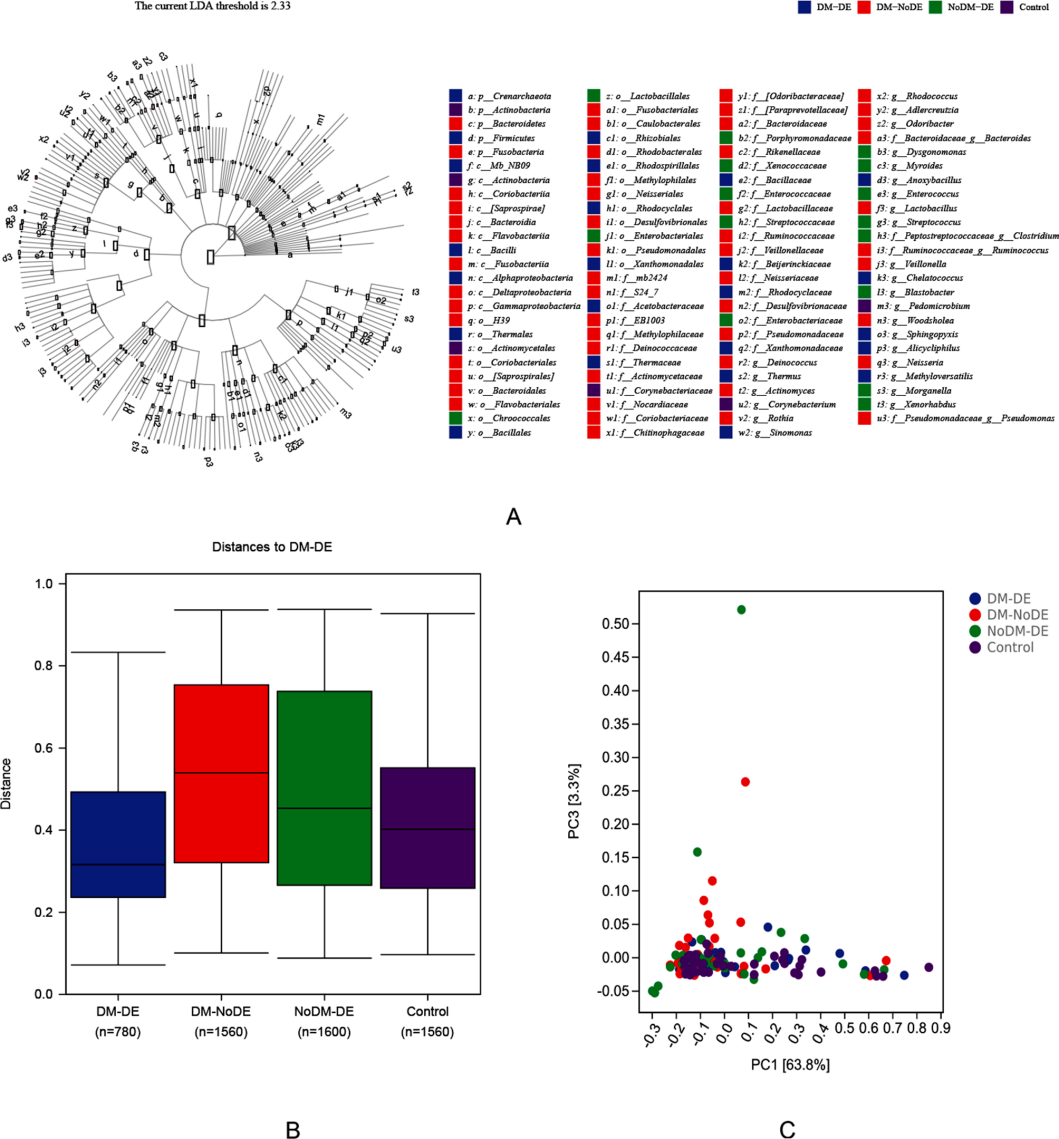
(**A**) LEfSe analysis in four groups; (**B**) analysis of differences between the four groups; (**C**) PCA analysis

LEfSe analysis in this study showed that abundance of *Firmicutes* in DM-DE group was significantly higher than that in other groups (*P* < 0.05); the abundance of *Bacteroidetes* in DM-NoDE group was significantly higher than that in other groups (*P* < 0.05). The abundance *Actinobacteria* in Control group was significantly higher than that in other groups (*P* < 0.05); The abundance of *Anoxybacillus* and *Chelatococcus* in DM-DE group were significantly higher than those in other groups (*P* < 0.05) > The abundance of *Pseudomonas* in DM-NoDE group was significantly higher than that in other groups (*P* < 0.05); and the abundance of *Staphylococcus* and *Clostridium* in NoDM-DE group were significantly higher than those in other groups (*P* < 0.05). The abundance of *Corynebacterium* in the control group was significantly higher than that in the other groups (*P* < 0.05) (Fig. 3A).

Using the Bray-Curtis distance matrix file and the python scikit-bio package (permanova), the difference analysis showed that there were statistically significant differences in species abundance between the DM-DE group and other groups in their conjunctival swabs (*P* < 0.05) (Table 2; Fig. 3B and C).

**Table 2:** Analysis of differences between the four groups

Group1	Group2	Samplesize	Permutations	pseudoF	p value	q value
All	-	158	999	5.211693	0.001*	-
DM-DE	DM-NoDE	79	999	12.492892	0.001*	0.002
DM-DE	NoDM-DE	80	999	6.279525	0.001*	0.002
DM-DE	Control	79	999	4.68895	0.004*	0.006

*P < 0.05

## Discussion

Our comprehensive review of literature, specifically focusing on PUBMED, revealed that prior research primarily concentrated on the ocular surface microbiome of diabetic dry eye patients within a clinical setting [22]. Remarkably, our study is the first to extend this research to the community-dwelling diabetic population, addressing a critical gap in the existing literature. Employing rigorous scientific methodologies and adhering to standardized definitions and protocols, our investigation uniquely incorporated the analysis of 16 S rRNA gene sequences from conjunctival swabs of community-based diabetic patients suffering from dry eye. This approach enabled us to not only confirm the presence of a distinct microbial signature associated with diabetic dry eye but also to elucidate the specific compositional dynamics of the core ocular microbiome in this demographic.

In our investigation, we observed notable distinctions in both the α and β diversity of the ocular surface microbiome (OSM) among diabetic patients with dry eye (DM-DE) and non-diabetic dry eye (NoDM-DE) subjects, in comparison to a normative cohort. These patient groups demonstrated elevated levels of both α and β diversity relative to the general population. This aligns with earlier studies which reported increased α diversity in OSM among dry eye (DE) patients without diabetes [29–32]. Our findings also corroborate with our prior research [22], which identified heightened α and β diversity in OSM among hospitalized diabetic patients with dry eyes, compared to a healthy control group. Shimizu et al. [32] have proposed that such variations could be linked to ocular surface epithelial damage and diminished mucin and antibacterial secretion in DE patients, potentially leading to a reduced clearance of ocular surface bacteria. Our earlier study [33], examining the ocular surface microbial composition in diabetic children with dry eye, revealed differences between diabetic and non-diabetic children. However, no significant disparity was noted in the microbial composition between diabetic children with and without dry eye. This suggests that the distinct pathogenesis of diabetes in children versus adults might influence the ocular surface’s microbial composition, with age maybe a potential contributing factor.

In this study, we discovered that about 98% of the ocular surface microbiome in the four examined groups predominantly belonged to four phyla: *Proteobacteria*, *Firmicutes*, *Actinobacteria*, and *Bacteroidetes*. Notably, the prevalence of *Firmicutes* was significantly higher than *Actinobacteria*. *Bacteroidetes* also emerged as a crucial component of the core ocular surface microbiome, with a broader range of microbial species identified compared to earlier reports [10–12, 15]. These variations in species composition and abundance might be attributed to our utilization of different sampling techniques and swab selections, alongside enhanced detection methods enabling the identification of a more diverse array of species. Our analysis indicated that *Firmicutes* were markedly more abundant in the DM-DE group than in other cohorts, while *Actinobacteria* predominated in the control group. This observation is in line with Wang Limin’s study [34] on ocular microbiome composition in type 2 diabetes. The noted differences in ocular microbiome between diabetic individuals and healthy subjects underscore the impact of blood glucose levels on ocular microbial composition. At the genus level, *Anoxybacillus* and *Chelococcus* were significantly more prevalent in the DM-DE group, with *Acinetobacter* and *Cupriavidus* also showing enrichment. The presence of *Anoxybacillus* on the ocular surface of the DM-DE group was notably higher. *Bacillus*, a well-studied, gram-positive, spore-forming bacterium, is typically found in the gut microbiome as either aerobes or facultative anaerobes. Their extensive range of secretory compounds can impact the integrity of the intestinal epithelial barrier. The potential translocation of chronic diseases like diabetes from the compromised gut to the eye has yet to be explored. However, the influence of gut flora alterations on ocular diseases and ocular flora shifts has been documented [35, 36]. The involvement of *Bacillus* and *Acinetobacter* in dry eye pathogenesis has also been reported [30]. In the NoDM-DE group, *Streptococcus* bacteria were more prevalent, aligning with the hypothesis that an increased abundance of *Streptococcus* may contribute to adult dry eye onset. Conversely, we found *Clostridium*, *Bacteroides*, *Lactobacillus*, and *Coprococcus* to be more abundant in the DM-NoDE group, a finding inconsistent with our previous study [22], which implicated unclassified *Clostridium* and *Lactobacillus* in adult diabetic dry eye onset. The disparity in ocular surface bacterial composition observed in this study could be linked to the older age demographic of our study population [37]. Additionally, the source of our research subjects differed; our previous study focused on hospital patients, whereas the current study was based on community screening. Factors such as the patient’s general condition and systemic diseases, environmental factors, lifestyle, exposure risk, medical history, dry eye severity, diabetes duration, blood sugar control, and other variables influencing the ocular microbiome were not accounted for the method of ocular sample collection also varied. Our previous study’s use of tear secretion test paper for detecting ocular surface microbiome differed significantly from the conjunctival swabbing employed in this study. The bactericidal components present in tears and conjunctival swabs, along with the differences in collection methods, likely contributed to the observed variations in ocular surface microbiome [38]. The dominant bacteria in the control group might represent a crucial element of the normal ocular surface microbiome, safeguarding the ocular microenvironment. However, the reduction and alteration of these dominant bacteria in DM-DE patients could be linked to the development and pathology of dry eye. The exact mechanisms by which these changes in the dominant ocular microbiome influence the onset and progression of dry eye remain to be elucidated.

Our study, however, is not without limitations. One significant constraint is that the current 16 S rRNA sequencing technology does not differentiate between live and dead bacteria [35, 36]. Additionally, the type and severity of dry eye were not considered in our analysis. The conjunctival swabbing method used for detecting ocular surface microbiome, being somewhat invasive, might affect the accuracy of the results, influenced by factors like swabbing depth and patient cooperation. Our assessment of the ocular surface microbiome, based on 98% homologous OTU clustering via Illumina MiSeq sequencing of bacterial 16 S rRNA genes, provided only relative abundance data of specific OTUs. For a more comprehensive understanding of microbial absolute abundance, future research might benefit from employing shotgun metagenomic sequencing or integrating standardized microbial communities. As a result, the specific functions and metabolic pathways of the ocular surface microbiome in dry eyes remain elusive, posing challenges in developing targeted therapeutic strategies for disturbances in ocular surface microecology.

## Conclusion

In summary, our research has illuminated significant shifts in the ocular surface microbiome (OSM) among diabetic patients suffering from dry eye. This study paves the way for future, more expansive research, including multicenter clinical trials with broader participant pools and repeated sampling to pinpoint bacterial species intricately linked to diabetic dry eye (DM-DE). Advancing to metagenomic sequencing methods would allow for a more comprehensive analysis, revealing not only the composition but also the functional aspects of the OSM in DM-DE cases. Such in-depth exploration is crucial for unraveling the complex pathogenesis of dry eye in diabetic patients. Ultimately, these insights hold the promise of enhancing our understanding of DM-DE at a molecular level and could be instrumental in crafting precise, effective treatment strategies.

### Electronic supplementary material

Below is the link to the electronic supplementary material.

Supplementary Material 1

## Data Availability

The detail data and materials in the current study are available from the SRA. database under the number PRJNA1048173 or use the following links (https://www.ncbi.nlm.nih.gov/sra/PRJNA1048173).
